# Low Serum Carotenoids Are Associated with Self-Reported Cognitive Dysfunction and Inflammatory Markers in Breast Cancer Survivors

**DOI:** 10.3390/nu10081111

**Published:** 2018-08-17

**Authors:** Krystle E. Zuniga, Nancy E. Moran

**Affiliations:** 1 Nutrition and Foods, Family and Consumer Sciences, Texas State University, 601 University Drive, San Marcos, TX 78666, USA; 2 United States Department of Agriculture/Agricultural Research Service Children’s Nutrition Research Center, Department of Pediatrics, Baylor College of Medicine, Houston, TX 77030, USA; nancy.moran@bcm.edu

**Keywords:** cancer-related cognitive impairment, cognition, carotenoid, memory, inflammation

## Abstract

**Background:** Dietary carotenoids may exert anti-inflammatory activities to reduce inflammation-driven cognitive impairments during cancer and cancer treatment. Our objective was to explore if cognitive function in breast cancer survivors (BCS) differs by serum carotenoid concentrations, and if blood carotenoids concentrations are associated with reduced systemic inflammation. **Methods:** Objective cognitive function and perceived cognitive impairment of 29 BCS and 38 controls were assessed cross-sectionally with the National Institutes of Health Toolbox Cognition Battery and The Functional Assessment of Cancer Therapy-Cognitive Function Questionnaire, respectively. Serum carotenoid and inflammatory marker (sTNF-RII, IL-6, IL-1ra, CRP) concentrations were measured. **Results:** Low-carotenoid BCS had more cognitive complaints compared to the low-carotenoid controls (Mdiff = −43.0, *p* < 0.001) and high-carotenoid controls (Mdiff = −44.5, *p* < 0.001). However, the cognitive complaints of high-carotenoid BCS were intermediate to and not different than the low-carotenoid BCS, or low- or high-carotenoid controls. BCS performed similarly to controls on all objective cognitive measures. Multiple linear regression, controlling for age and body mass index (BMI), demonstrated an inverse association between serum carotenoid concentrations and pro-inflammatory sTNFR-II (β = 0.404, *p* = 0.005) and IL-6 concentrations (β = −0.35, *p* = 0.001), but not IL-1ra or CRP. **Conclusions:** Higher serum carotenoid concentrations may convey cognitive and anti-inflammatory benefits in BCS. Future research should identify dietary components and patterns that support cognitive health in cancer survivors.

## 1. Introduction

With over 252,000 new breast cancer cases estimated in 2017 and a 90% five-year survival rate, the population of over 3.5 million U.S. breast cancer survivors (BCS) will continue to increase [1,2]. BCS live with many treatment-related side effects including cognitive dysfunction [3]. There is evidence that up to 75% of breast cancer patients experience cognitive decline during cancer treatment and that these impairments can persist up to twenty years following treatment completion [4,5]. However, there is marked variability in the incidence, duration, and severity of cancer-related cognitive impairments, suggesting that risk is modulated by treatment type, demographics, lifestyle, or genetics [4]. Recent advances in the field of nutritional neuroscience suggest that diet may play a role in preventing aging-associated cognitive decline; however, little to no research has focused on the mechanisms by which dietary factors reduce or prevent cancer-related cognitive decline. 

Cancer-related cognitive impairments are hypothesized to arise as a result of cancer- and treatment-related inflammation. Cytokine elevation occurs in breast cancer patients before and during chemotherapy [6] and persists post-treatment [7,8,9]. These cytokines may be stimulated by the tumor, treatment, or psychological factors like fatigue or depression [10]. In breast cancer patients, elevated cytokines have been associated with reduced cognitive performance, changes in perceived cognitive function, and smaller hippocampal volume [10]. Oxidative stress and inflammation adversely affect neurogenesis and have a role in the pathogenesis of cognitive dysfunction [10], making these important target mechanisms to investigate with regard to the cognitive side effects of cancer treatment.

Nutrition has an important role in brain structure and function; for example, dietary components maintain neural tissue and membrane structure, provide substrates for signaling molecule synthesis, fuel metabolism, and some have antioxidant and anti-inflammatory actions [11]. Evidence suggests a correlation between fruit and vegetable intake and cognitive function [12,13], and fruits and vegetables are components of dietary patterns associated with reduced risks of cognitive impairment and neurodegenerative diseases [14,15,16]. In a prospective study of over 1000 breast cancer patients, both fruit and vegetable intake were associated with better verbal fluency scores post-diagnosis [17]. We previously discovered a positive relationship between fruit and vegetable intake and a measure of executive function (the ability to focus on relevant aspects of the environment) among BCS and age-matched controls [18]; however, the mechanisms underlying this relationship remain unknown. While fruits and vegetables contain an abundance of nutrients and bioactives, carotenoids, which are particularly concentrated in fruits and vegetables [19], have recently gained attention for potential cognitive benefits across the lifespan [20,21,22]. In addition to some carotenoids serving as vitamin A precursors, carotenoids confer many physiological activities, such as acting as anti-inflammatory or antioxidant agents, and may exert benefits for cognitive health, as some carotenoids may have potent activity in scavenging free radicals, protecting lipid membranes from peroxidation and reducing cellular inflammation [19,23,24]. A number of recent studies have suggested a particular benefit of dietary carotenoid intake for attenuating the symptoms of aging-related cognitive decline [25,26,27,28].

Some inflammatory and oxidative processes associated with cognitive decline in aging are believed to be similar to those that occur in cancer-related cognitive impairment; therefore, we hypothesize that potential anti-inflammatory and neuroprotective actions of carotenoids may elicit cognitive benefits in BCS. It is of interest to determine if the benefits of carotenoids to cognition and antioxidant status observed among a healthy population would be seen in other populations at risk of cognitive decline such as BCS. The objective of this research is to explore if cognitive function in BCS differs depending on their serum carotenoid concentrations and to explore reduction of inflammation as a potential mechanism by which carotenoids are associated with cognitive function in BCS.

## 2. Materials and Methods 

Recruitment: Breast cancer survivors who completed primary treatment (chemotherapy, radiation therapy, or both) within the past 60 months and age-matched controls with no history of a cancer diagnosis were recruited from the Central Texas area. Potential participants were recruited via local oncology clinics, support groups, print media (flyers), social networking sites, websites, and listserv announcements. After expressing initial interest, individuals were contacted by phone and provided with a full study description and screened for eligibility. Inclusion criteria included: female; breast cancer survivor ≤60 months from last treatment, 30–70 years old; no history of stroke, heart attack, or transient ischemic attack; not currently pregnant; could speak, read, and write English; could attend all testing sessions; not blind or legally blind; nonsmoker. The exclusion criteria for this study included: current use of computer-based brain training games (e.g., Lumosity^®^, BrainHQ^®^). Eligible participants were scheduled for two testing appointments. Of the 83 total contacts, 70 consented and were eligible for testing (30 cancer survivors, 40 controls). Three women withdrew after consenting and did not complete testing owing to being no longer interested or no longer eligible (1 cancer, 2 control); thus, data are presented from sixty-seven women that consented and completed testing (38 control; 29 cancer). Participants were remunerated for their participation. All study procedures and recruitment materials were approved by the Texas State University Institutional Review Board and were performed in accordance with the ethical standards as laid down in the 1964 Declaration of Helsinki and its later amendments. Written informed consent was obtained from all subjects. This trial was registered at ClinicalTrials.gov as NCT02591316.

Demographics: Participants self-reported medical history, marital status, age, race, ethnicity, occupation, income, and education level. Additionally, BCS self-reported breast cancer specific diagnosis and treatment history. Height and weight were measured to calculate body mass index (BMI). 

Dietary Intakes: Participants completed a modified version of the 2005 Block Food Frequency Questionnaire [29] reporting on intakes over the past 3 months delivered via NutritionQuest’s online Data-on-Demand System. The questionnaire included 110 food items with details on portion sizes.

Neuropsychological assessment: The computer-based National Institutes of Health Toolbox Neurological and Behavioral Function Cognitive Function Battery (NIHTB-CB) was used to assess multiple domains of cognitive function. The NIHTB-CB has high reliability and validity and has been widely used in various research applications to assess cognitive performance [30]. Episodic memory and working memory were tested by the picture sequence and list sorting working memory tasks, respectively. Picture Vocabulary and Oral Reading Recognition tested language abilities. Fully-adjusted scores for each participant were provided by the NIHTB-CB and are normalized to reference groups by age, ethnicity, gender, and education. 

Self-reported cognitive dysfunction: Perceived cognitive function was measured with the Functional Assessment of Cancer Therapy-Cognitive Function (FACT-Cog), a 37-item, 5-pt Likert scale questionnaire [31]. Possible scores range from 0–148, with higher FACT-Cog scores indicating fewer cognitive complaints. In this study, the Cronbach alpha was 0.98 for the total score, with subscale scores ranging from 0.91 to 0.96.

Inflammatory markers: Participants were instructed to fast for 10 h prior to blood collection. Fasted blood samples were collected via venipuncture into silica-coated BD Vacutainer serum collection tubes. Samples were allowed to clot for 30 min, centrifuged for 15 min at 2000× *g*, and the resultant serum was aliquoted and stored at −80 °C for subsequent analysis. Serum C-reactive protein (CRP), soluble tumor necrosis factor-alpha receptor type II (sTNF-RII), and interleukin-6 (IL-6) were quantified using their respective Quantikine ELISA Immunoassay kit (R&D Systems, Minneapolis, MN) per the manufacturer’s instructions. Interleukin-1 receptor antagonist (IL-1ra) was analyzed with Biosource IL-1ra Cytoscreen kit (Biosource Europe S.A., Nivelles, Belgium) per the manufacturer’s instructions.

Serum carotenoids: All sample analyses were performed under yellow lighting to protect carotenoids. Carotenoids were extracted from 300 uL thawed serum based on a previously described method with several modifications [32]. Apo-8’-carotenal (75 ng, #10810, Sigma-Aldrich, St. Louis, MO, USA) was added as an internal standard to duplicate samples from each subject. Dried extracts were reconstituted in 30 µL 80% methyl *tert*-butyl ether (MtBE): 20% methanol, held at 4 °C in a refrigerated autosampler, and 20 µL were injected onto an Ultimate 3000 UHPLC system coupled with a photodiode array detector (ThermoFisher, Waltham, MA, USA). Analytes were separated on a C30 Carotenoid Column (250 × 3 mm, 3 µm particle size) (ThermoFisher, Waltham, MA, USA ) cooled to 18 °C, over 42 min at 0.4 mL/min by a gradient elution method, which was a modification of a previously published method [33]. The method utilized 3 HPLC-grade mobile phases: A: Methanol, B: MtBE, C: 1.5% Ammonium Acetate in water (*w/v*). The initial conditions were 98% A, 0% B, 2% C, which reached to 60% A, 38% B, 2% C at 12.5 min, then 48% A, 50% B, and 2% C at 20 min, then 38% A, 60% B, and 2% C at 30 min, which was held until 32 min, then returned to initial conditions (98% A, 0% B, 2% C) by 35 min and was held at initial conditions until 42 min. Analytes were detected by photodiode array detector at 472 nm (lycopene, beta-apo-8’-carotenal), 290 nm (phytoene), 450 nm (alpha- and beta-carotene, lutein & zeaxanthin, and beta-cryptoxanthin), and 330 nm (phytofluene). The data were analyzed using Chromeleon 7 software (ThermoFisher, Waltham, MA, USA). Analyte signals were quantitated by external calibration curves of authentic analytical standards for (*E/Z*)-phytoene, lycopene, beta-carotene, alpha-carotene, lutein, zeaxanthin, and beta-cryptoxanthin (Sigma-Aldrich, St. Louis, MO, USA), phytofluene (Carotenature, Lupsingen, Switzerland), and results were adjusted to the internal standard to correct for extraction efficiency. Total carotenoid concentration was the sum of all carotenoids analyzed.

Plasma Cholesterol: Samples were analyzed by commercial lab (LabCorp, Houston, TX, USA) using the lipid panel test (#303756) for frozen, EDTA-collected plasma. Triglycerides and total and HDL cholesterol were measured directly by enzymatic colorimetric assay on a clinical chemistry analyzer (COBAS 8000 c701, Roche Diagnostics International Ltd., Rotkreuz, Zug Switzerland) and VLDL and LDL cholesterol were calculated using standard clinical algorithms.

Statistical Analysis: The primary objective of this study was to determine if cognitive function in BCS differed by serum total carotenoid status. We also confirmed that serum carotenoid concentrations were positively associated with reported fruit and vegetable intake. As secondary outcomes, we explored whether serum carotenoids were associated with markers of inflammation. Characteristics of the BCS and controls were compared using independent t-tests for continuous variables and *Χ^2^*-tests for categorical variables. Differences in serum carotenoids between groups were tested with the Mann–Whitney U-test. Univariate analysis of covariance (ANCOVA) was used to determine the difference in subjective cognitive function between BCS and controls after controlling for age. In order to compare cognitive function between BCS and controls with either low or high serum carotenoid concentrations, BCS and controls were each split into two groups: (1) BCS or control with serum carotenoid concentrations lower than, and including, the median; and (2) BCS or control with serum carotenoid concentrations above the median. Then, univariate ANCOVA, including age as a covariate, was used to compare cognitive function as a function of group (low carotenoid BCS, high carotenoid BCS, low carotenoid control, high carotenoid control), and post hoc analysis with *t*-tests was performed with a Bonferroni adjustment for the six comparisons, yielding an α = 0.008. The significance level was set at α = 0.05 for all other statistical analyses. Associations between serum carotenoids and inflammatory markers were determined using multiple linear regression, controlling for the a priori-identified covariates of age and BMI. All statistical analyses were performed in SPSS (v.24) (IBM, Armonk, NY, USA). When the assumption of normality was violated, values were log- transformed to the base 10 (log_10_) to provide increased normality. 

## 3. Results

**Participant Characteristics:** There were no significant differences in age, education, race, ethnicity, socioeconomic status, or BMI between BCS and healthy controls (Table 1). Most BCS had early stage breast cancer at diagnosis (75.9% at stage II or lower) and were an average of 1.6 years post-completion of cancer treatment (M = 18.6 months, SD = 16.3). All had undergone surgery, 65.5% had received radiation therapy, 72.4% had received chemotherapy, and 65.5% were currently receiving hormonal therapy.

**Dietary and serum carotenoids:** Serum and dietary carotenoids were not significantly different between BCS and controls (Table 1). Fruit and vegetable intake were similar between BCS and age-matched controls (Table 1). After controlling for BMI and total cholesterol, reported fruit and vegetable intake (total servings per day) was positively correlated with total serum carotenoid concentrations (*r* = 0.434, *p* < 0.001) (data not shown).

**Objective and Subjective Cognitive Function:** BCS performed similarly to controls on all objective cognitive measures (Table 2). However, BCS reported significantly more cognitive complaints than controls (Table 3). After adjustment for age, there was a statistically significant difference in perceived cognitive function between the groups across all subscales and total score of the FACT-Cog, indicating more self-reported cognitive difficulties in BCS (Table 3). 

**Figure 1:**
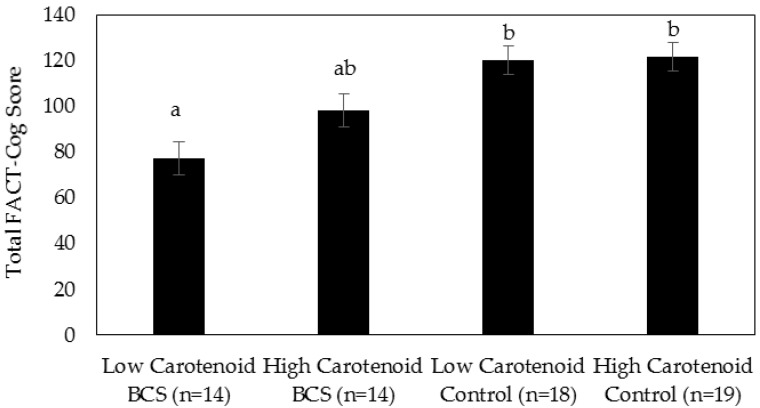
Perceived Cognitive Dysfunction by Group Carotenoid Status. Data are adjusted mean (standard error). ANCOVA with age as covariate. Different letters indicate significant differences between groups (*p* < 0.05).

**Serum carotenoids and cognitive complaints:** Serum carotenoid concentrations by group are described in Table 4. ANCOVA was used to determine the differences in FACT-Cog scores between serum carotenoid status groups. After adjustment for age, there was a statistically significant difference in FACT-Cog scores between the groups, F (3, 60) = 9.498, *p* < 0.001, partial η2 = 0.322. The low-carotenoid BCS had significantly lower FACT-Cog scores (more cognitive complaints) compared to the low-carotenoid controls (Mdiff = −43.0, 95% CI [−68.8, −17.1], *p* < 0.001) and high-carotenoid controls (Mdiff = −44.54, 95% CI [−71.1, −18.0], *p* < 0.001) (Figure 1). However, FACT-Cog scores in high-carotenoid BCS were not significantly different than those of the low-carotenoid BCS, or low- or high-carotenoid controls.

**Inflammatory Markers:** Serum CRP, sTNF-RII, IL-6, and IL-1ra levels were not significantly different between BCS and controls (Table 5). In multiple linear regression models controlling for age and BMI, serum carotenoids were significant predictors of lower IL-6 (β = −0.353, *p* = 0.001) and sTNFR-II (β = −0.404, *p* = 0.005) concentrations, but not IL-1ra or CRP (Table 6). 

## 4. Discussion

Here we report, for the first time, that BCS with low blood carotenoid concentrations reported more cognitive complaints than healthy controls with no prior cancer diagnosis, and BCS with higher serum carotenoid concentrations reported cognitive complaints not different from both groups of controls. Furthermore, we found that several, but not all, serum markers of inflammation were significantly inversely associated with serum total carotenoid concentrations, suggesting a potential mechanism by which carotenoids may affect cognitive function. 

In our sample, objectively assessed cognitive performance was similar between BCS and controls. Indeed, several meta-analyses have concluded that cognitive deficits in BCS are generally subtle [34,35,36], and may be more detectable by subjective report than by objective laboratory tests. Standard neuropsychological assessments likely command more focus than the tasks of everyday life and may not be representative of the routine cognitive challenges that BCS face [37,38]. Compared to age-matched controls, BCS reported significantly greater levels of perceived cognitive impairment. In non-cancer populations, memory complaints are a predictor of worse physical health and a higher degree of dependency for daily life activities [39,40]. In aging populations, self-reported, perceived memory impairments are associated with an increased risk of cognitive decline and impairment and all-cause dementia, suggesting that cognitive complaints may indicate neurodegenerative changes that precede significant changes in neuropsychological performance assessed with objective laboratory tests [41,42,43]. A minimal clinically important difference for FACT-Cog has been estimated to be 7–11 points [44] and has been demonstrated to be sensitive to interventions to improve cognitive function in cancer survivors including cognitive rehabilitation [45], physical activity [46,47], and meditation [48]. The low carotenoid BCS had an over 40 point difference on FACT-Cog scores compared to low and high carotenoid controls, which is clinically significant. 

The neurotoxicity of cancer and treatment may be mediated, at least in part, through the action of cytokines crossing the blood-brain barrier to elicit oxidative stress and negatively impact neurogenesis [49]. Evidence suggests that several inflammatory markers are elevated in BCS including the cytokines IL-6, IL-8, and IL-10 [7,8,9,10]; however, BCS in our sample did not have significantly higher levels of inflammatory markers compared to controls. This may have been partially attributed to the heterogeneity in time since treatment in our small sample size, as it is suggested that some cytokine changes may be acute [8,50]. 

Carotenoids can accumulate in the brain and may function as reactive oxygen species scavengers, protecting lipid membranes from peroxidation and reducing cellular inflammation [23,51,52]. Evidence indicates an altered redox status in cancer patients [53,54], and other lines of evidence suggest oxidative stress in the brain can negatively affect neurogenesis and promote cognitive dysfunction [55,56]. However, the association between biomarkers of oxidative stress in cancer survivors and cancer-related cognitive dysfunction has not been thoroughly assessed, and future research is needed to determine which oxidative stress markers have clinical and prognostic utility in this sample. The current analyses focused on inflammatory markers that have consistently been identified to be both elevated and correlated with cognitive function in breast cancer patients and survivors. In this study, serum carotenoids were inversely associated with IL-6 and sTNFR-II, which is notable, as elevations in these markers of systemic inflammation have been associated with alterations in brain structure and function in breast cancer patients and survivors. sTNF-RII is a marker of the cytokine TNF-α’s pro-inflammatory activity and can be reliably measured in circulation [57]. Higher sTNF-RII levels have been associated with greater memory complaints [50], worse memory performance [58], alterations in brain metabolism [50], and decreased gray matter volume in specific brain regions [59] of BCS. Additionally, a significant correlation between lower hippocampal volume and higher TNF-α has been observed among BCS [60]. In a longitudinal sample of BCS, elevated IL-6 levels were associated with more cognitive complaints [9], and in a sample of breast cancer patients, impaired memory function by radiation therapy was suggested to be partially mediated by elevated IL-6 [61]. The precise mechanism underlying how peripheral inflammatory cytokines impairs cognitive function in BCS is unknown. However, it is hypothesized that peripherally elevated levels of cytokines induce alterations among numerous neural substrates, including neurotransmitters and brain-derived neurotrophic factor [10]. It is mechanistically plausible that carotenoids have a direct effect on systemic inflammation, as carotenoids have been shown in model systems to reduce oxidative stress and downstream inflammatory signaling (reviewed in Reference [62]). In BCS, higher plasma carotenoid concentrations have previously shown to be associated with reduced oxidative stress [63,64]. Therefore, strategies to reduce inflammation, such as dietary changes to increase carotenoid intake, could provide cognitive benefits. 

In addition to being bioactive compounds themselves, serum concentrations of carotenoids are a well-recognized biomarker of fruit and vegetable intake [65]. In our sample, serum carotenoid concentrations were positively associated with fruit and vegetable intake. Fruits and vegetables contain an array of antioxidants and anti-inflammatory nutrients and bioactives that may elicit neuroprotection by inhibiting neuroinflammation and neurodegeneration [66,67]. The carotenoids lutein and zeaxanthin have been more strongly associated with cognitive function than other individual carotenoids [22]. Further investigation is warranted to determine which specific compounds contribute to cognitive benefits observed with fruit and vegetable consumption, as many compounds likely interact to elicit cognitive benefits. The limited evidence of interventions with fruits and vegetables or carotenoids for improvement in cognitive function in aging populations does suggest a positive benefit [25,26,27,28,66,68,69]; thus, promotion of this dietary strategy may be useful for reducing the short- and/or long-term adverse cognitive effects of cancer treatment. 

There are important limitations of this study including the cross-sectional design and small sample size which limits the interpretation of our findings. Our sample of BCS was heterogeneous in cancer stage, time since diagnosis, and treatment variables. The neuropsychological assessment was brief (~40 min), thus, a longer battery or field testing may have identified the subtle cognitive impairments common in BCS. There are several other inflammatory markers that were not assessed; however, CRP, IL-6, sTNF-RII, and IL-1ra were chosen because previous studies have demonstrated these markers to be elevated and correlated with cognitive decline in BCS [50,60,70]. While we cannot directly measure brain oxidative stress or inflammation, evidence indicates systemic inflammation is associated with lower white matter integrity, poorer cognitive performance, dementia, and cognitive decline in aging [71,72]. Future basic research can define the impact of carotenoids on other proposed mechanisms of cognitive dysfunction requiring model systems like oxidative stress, epigenetic changes, impaired neurogenesis, and treatment-induced hormonal alterations [4]. Despite these limitations, this is the first study, to our knowledge, to examine a potential mechanism by which food components could elicit cognitive benefits in cancer survivors. Although previous research has supported the hypothesis that dietary carotenoids have anti-inflammatory activity and may elicit cognitive benefits, these associations have never been explored in individuals with cancer-related cognitive impairment. Only two studies have examined the association between dietary intake and cognitive dysfunction in cancer survivors [17,18], and neither explored a potential mechanism of cognitive benefits from dietary components. Therefore, our findings are novel and provide much needed preliminary information to guide future dietary interventions. 

Cancer survivorship research to date has primarily focused on improving survival and reducing risk of recurrence, yet strategies to support survivors’ cognitive function are limited. Pharmacotherapies modestly alleviate cognitive deficits [73,74]; however, possible side effects and interactions highlight a need to explore modifiable lifestyle factors. While cognitive behavioral training and physical activity convey functional improvements in cancer survivors [75,76], there are no published dietary strategies. Nevertheless, survivors seek dietary changes to improve symptoms and reduce cancer risk [77]. Our findings suggest an association between serum carotenoids, perceived cognitive function, and markers of systemic inflammation. Future research should explore the mechanisms by which dietary patterns and corresponding food components can reduce memory complaints and support cognitive health in cancer survivors, providing evidence for future dietary interventions to improve BCS’ quality of life. 

## Figures and Tables

**Table 1:** Participant Characteristics.

	Breast Cancer Survivors	Controls	*p*
Age, y (Mean ± SD)	50.1 ± 10.1	50.8 ± 10.0	0.783
Income, *n* (%)			
≥$60,000	16 (61.5)	29 (78.4)	0.145
Race, *n* (%)			
White	20 (69.0)	33 (86.8)	0.075
Black	3 (10.3)	0 (0.0)	
Asian	2 (6.9)	1 (2.6)	
More than One Race	4 (13.8)	4 (10.5)	
Ethnicity, *n* (%)			
Hispanic or Latino	6 (20.7)	9 (23.7)	0.771
Education, *n* (%)			
≥ 4 year College Degree	20 (69.0)	32 (84.2)	0.138
Body Mass Index (BMI), kg/m^2^ (Mean ± SD)	29.7 ± 6.3	27.3 ± 7.6	0.168
Stage at Diagnosis, *n* (%)			
Ductal Carcinoma in Situ (DCIS)	3 (10.3)	-	
Stage I	8 (27.6)	-	
Stage II	11 (37.9)	-	
Stage III	6 (20.7)	-	
Unknown	1 (3.4)	-	
Treatment, *n* (%)			
Chemotherapy Only	10 (34.5)	-	
Radiation Only	8 (27.6)	-	
Chemotherapy + Radiation	11 (37.9)	-	
Current Hormone Therapy	19 (65.5)	-	
Surgery	29 (100)	-	
Time since Treatment-Months (Mean ± SD)	18.6 ± 16.3	-	
Total Cholesterol (mg/dL) ^1^	167 (130–189)	166 (146–189)	0.463
Serum Carotenoid Concentrations (nmol/L) ^1^			
Alpha-carotene	41.1 (19.7–64.5)	53.3 (35.8–122.7)	0.121
Beta-carotene	163.0 (79.5–298.8)	217.5 (122.6–367.2)	0.260
Lycopene	298.1 (225.8–369.8)	312.4 (213.6–411.3)	0.968
Lutein & Zeaxanthin	163.2 (92.9–272.9)	214.7 (145.7–308.3)	0.199
Beta-cryptoxanthin	68.7 (45.3–115.8)	78.2 (49.6–154.9)	0.354
Phytofluene	65.2 (50.7–92.4)	62.1 (48.7–100.2)	0.958
Phytoene	68.6 (50.3–82.4)	53.6 (43.1–75.6)	0.083
Total Carotenoids	933.3 (663.2–1120.5)	1052.3 (782.2–1356.1)	0.314
Average Daily Intakes (Mean ± SD)			
Fruit (cups)	1.0 ± 0.8	1.1 ± 0.9	0.568
Vegetables (cups)	2.8 ± 1.7	2.6 ± 1.6	0.761
Total Carotenoid Intake (mg)	20.8 ± 13.5	18.7 ± 10.3	0.514

^1^ median and interquartile range (25–75th percentile).

**Table 2:** Objective Cognitive Function ^1^.

	Breast Cancer Survivors	Controls	*p*
List-Sorting Working Memory	108.8 (17.5)	105.3 (11.2)	0.353
Picture Vocabulary	108.2 (16.2)	115.2 (14.5)	0.072
Picture Sequence Memory	100.5 (18.9)	103.1 (14.7)	0.536
Oral Reading Recognition	117.4 (14.3)	116.7 (16.4)	0.864

Mean (SD).^1^ fully-adjusted, standardized scores.

**Table 3:** Self-Reported Cognitive Dysfunction.

	Breast Cancer Survivors	Controls	*p*
Total FACT-Cog Score ^1^	88.70 (5.27)	119.17 (4.60)	<0.001
Perceived Cognitive Impairments	45.43 (3.01)	61.66 (2.63)	<0.001
Impact of Perceived Cognitive Impairments on Quality of Life	10.06 (0.83)	13.60 (0.73)	0.002
Comments from Others	12.94 (0.53)	15.57 (0.46)	<0.001
Perceived Cognitive Abilities	20.28 (1.44)	28.34 (1.26)	<0.001

Data are adjusted mean (SE). ANCOVA with age as a covariate. ^1^ Functional Assessment of Cancer Therapy-Cognitive Function

**Table 4:** Serum Carotenoid Concentrations.

Serum Carotenoid Concentrations (nmol/L).	Low Carotenoid BCS	High Carotenoid BCS	Low Carotenoid Control	High Carotenoid Control
Alpha-carotene	27.3 (13.0)	153.8 (269.9)	43.3 (33.6)	90.3 (150.8)
Beta-carotene	103.9 (58.7) ^a^	461.1 (427.4) ^b^	140.7 (78.5) ^a^	425.4 (215.7) ^b^
Lycopene	275.0 (74.4) ^a^	344.1 (99.4) ^ab^	241.4 (80.8) ^a^	417.3 (198.4) ^b^
Lutein & Zeaxanthin	117.4 (44.5) ^a^	366.3 (263.6) ^b^	174.0 (79.5) ^ac^	299.8 (121.7) ^bc^
Beta-cryptoxanthin	54.4 (34.5) ^a^	135.9 (133.5) ^ab^	69.1 (58.1) ^ab^	149.4 (105.3) ^b^
Phytofluene	55.5 (34.6) ^ab^	113.8 (91.8) ^a^	53.9 (22.5) ^b^	108.4 (63.4) ^a^
Phytoene	56.5 (23.1) ^ab^	87.5 (40.1) ^a^	47.9 (9.9) ^b^	79.7 (39.0) ^a^
Total Carotenoids	689.9 (185.0) ^a^	1662.4 (1065.7) ^b^	770.3 (179.9) ^a^	1619.6 (604.2) ^b^

Mean (SD); Different letters indicate significant differences between groups (*p* < 0.05).

**Table 5:** Inflammatory Markers.

	Breast Cancer Survivors	Controls	*p*
IL-6 (pg/mL)	2.0 (1.1)	1.8 (1.1)	0.431
IL-1ra (pg/mL)	476.8 (91.6)	513.1 (78.4)	0.766
sTNFRII (pg/mL)	3037.41 (128.0)	2717.3 (115.8)	0.073
CRP (ng/mL)	2156.8 (358.2)	3012.3 (296.3)	0.072

Data are mean adjusted (SE). ANCOVA with age, BMI, and moderate to vigorous physical activity as covariates.

**Table 6:** Regression analyses explaining variability in inflammatory markers.

	IL-6			sTNFR-II			CRP			IL-1ra		
	*β*	*p*	Model Adjusted *R^2^*	*β*	*p*	Model Adjusted *R^2^*	*β*	*p*	Model Adjusted *R^2^*	*β*	*p*	Model Adjusted *R^2^*
			0.483 **			0.175 *			0.142 *			0.032
Age	0.225	0.020		0.284	0.022		−0.064	0.622		0.264	0.046	
BMI	0.433	<0.001		0.036	0.790		0.337	0.024		−0.018	0.900	
Serum Carotenoids	−0.353	0.001		−0.404	0.005		−0.158	0.277		−0.162	0.267	

* *p* < 0.01, ** *p* < 0.001.
